# Mind-Body Medicine in the Treatment of Depression: A Narrative Review of Efficacy, Safety and Mechanisms

**DOI:** 10.1007/s11920-024-01548-7

**Published:** 2024-10-19

**Authors:** Hazal Sarak Kucukosmanoglu, Holger Cramer, Rahele Tavakoly, Alina Moosburner, Mirela-Ioana Bilc

**Affiliations:** 1 Institute of General Practice and Interprofessional Care, University Hospital Tübingen, Osianderstr. 5, Tübingen, 72076 Germany; 2 Robert Bosch Center for Integrative Medicine and Health, Bosch Health Campus, Stuttgart, Germany; 3 Gulhane Faculty of Physiotherapy and Rehabilitation, University of Health Sciences, Ankara, Turkey

**Keywords:** Mind body therapies, Mind body medicine, Depressive disorders, Depressive symptoms, Mental health

## Abstract

**Purpose of the Review:**

This narrative review examines the efficacy, mechanisms and safety of mind-body medicine (MBM) in the treatment of depression. We reviewed the potential effects of various MBM interventions such as yoga, tai chi, qigong, mindfulness-based interventions and nutrition on clinical and subthreshold depressive symptoms.

**Recent Findings:**

Current studies indicate a growing interest in the use of MBM for psychiatric disorders, including depression. MBM interventions demonstrate efficacy in reducing depressive symptoms with fewer adverse effects and costs compared to pharmacological treatments.

**Summary:**

MBM has significant potential to improve mental health outcomes for depression. These interventions encourage self-care and stress management through behavioural, exercise, relaxation and nutritional approaches. While existing data are promising, further, more rigorous studies are required to confirm long-term effectiveness and to determine the role of MBM in comprehensive depression treatment strategies.

**Supplementary Information:**

The online version contains supplementary material available at 10.1007/s11920-024-01548-7.

## Introduction

Depression has become one of the most common mental health problems in the world, affecting approximately 280 million people with a prevalence rate of about 3.4% worldwide [1]. In light of this global increasing trend [2], prevention and treatment of depression requires effective interventions, including modification of risk factors [3] such as changes in the release of certain chemicals in the brain, genetic factors, personality traits, and environmental factors [4].

While depression can manifest across all demographic groups, higher prevalence rates have been observed in specific sub-samples such as the elderly [5, 6], women in general as well as women who are pregnant or have recently given birth [7, 8] and among college and university students in early adulthood [9, 10].

Major depressive disorder (MDD) is one of the most common forms of depressive disorder [11]. Patients can experience one or more major depressive episodes, defined as two weeks or more of depressed mood/or loss of interest or enjoyment and at least four additional symptoms [12]. On the other hand, subthreshold depression is defined as an umbrella term that covers a variety of conditions that do not meet the criteria for depressive disorder but negatively affect quality of life and are not related to another condition [4, 13, 14]. Other forms are late-life depression (LLD), and perinatal depression including pre- and postnatal depression [12, 15–18].

Psychotherapy, medication or a combination of both are used as standard treatments for depression. Although about 20–40% of people with depressive disorders generally respond well to pharmacological treatments and psychotherapy, many are resistant to treatment [19], and some have limited access to treatment. This situation creates a major challenge for the medical system and brings enormous medical costs and economic burdens to patients and society [20, 21]. The large treatment gap between those needing and receiving mental health treatment is a social problem, especially in developing countries and lower socioeconomic groups [22, 23]. Resistance to pharmacotherapy in depression may lead to decreased drug efficacy depending on the genetic characteristics of individuals (differences in the enzymes that metabolize antidepressants) [24]. In addition, HPA axis dysregulation due to high cortisol levels and high levels of inflammatory markers such as C-reactive protein (CRP) may impair antidepressant responses in depressed patients [25]. Socio-economic difficulties, unemployment, poor working conditions, limited access to mental health services, cultural differences especially in rural and conservative areas may reduce the effectiveness of both pharmacological treatment and psychotherapy [26–28]. Depressive patients may sometimes resist pharmacotherapy and psychotherapy due to beliefs that their condition stems not from biochemical imbalances, but from spiritual, social, or existential disturbances [29]. They may also fear the side effects of medications, leading to poor adherence or treatment avoidance [30]. According to the non-medical health belief model, individuals may prefer complementary and integrative approaches or lifestyle changes over conventional medical interventions, and recognizing and respecting these beliefs is a key aspect in the relationship between patients and health care providers, especially in a cross-cultural context [31].

In this way, complementary and integrative treatments such as mind-body medicine (MBM) offer a potential solution to the shortage of available treatment alternatives [32, 33]. MBM is an integrative approach that focuses on the interconnectedness of the brain, mind, body and behaviour, using techniques that promote self-awareness and self-care and support the mind’s capacity to influence physical health and bodily functions [34]. MBM aims to improve health by focusing on the individual and taking into account individual capabilities [35]. It acknowledges the ways in which emotional, mental, social, spiritual, and behavioural factors can directly impact health, and tries to make use of them [36]. There are many practices that are used as MBM techniques. These approaches encompasses various modalities, including relaxation, meditation, and guided imagery, which share commonalities with manual, breathing and movement therapies that incorporate mindful practices such as yoga, tai chi and qigong. In addition, a broader perspective can be considered exploring mindful practices within other treatment modalities such as hypnotic suggestions [37], acupuncture [38], ayurvedic massage and anthroposophic nursing techniques such as rhythmic massage [14, 39]. While acknowledging this broader view, including these practices is out of the scope of this review.

**Fig. 1:**
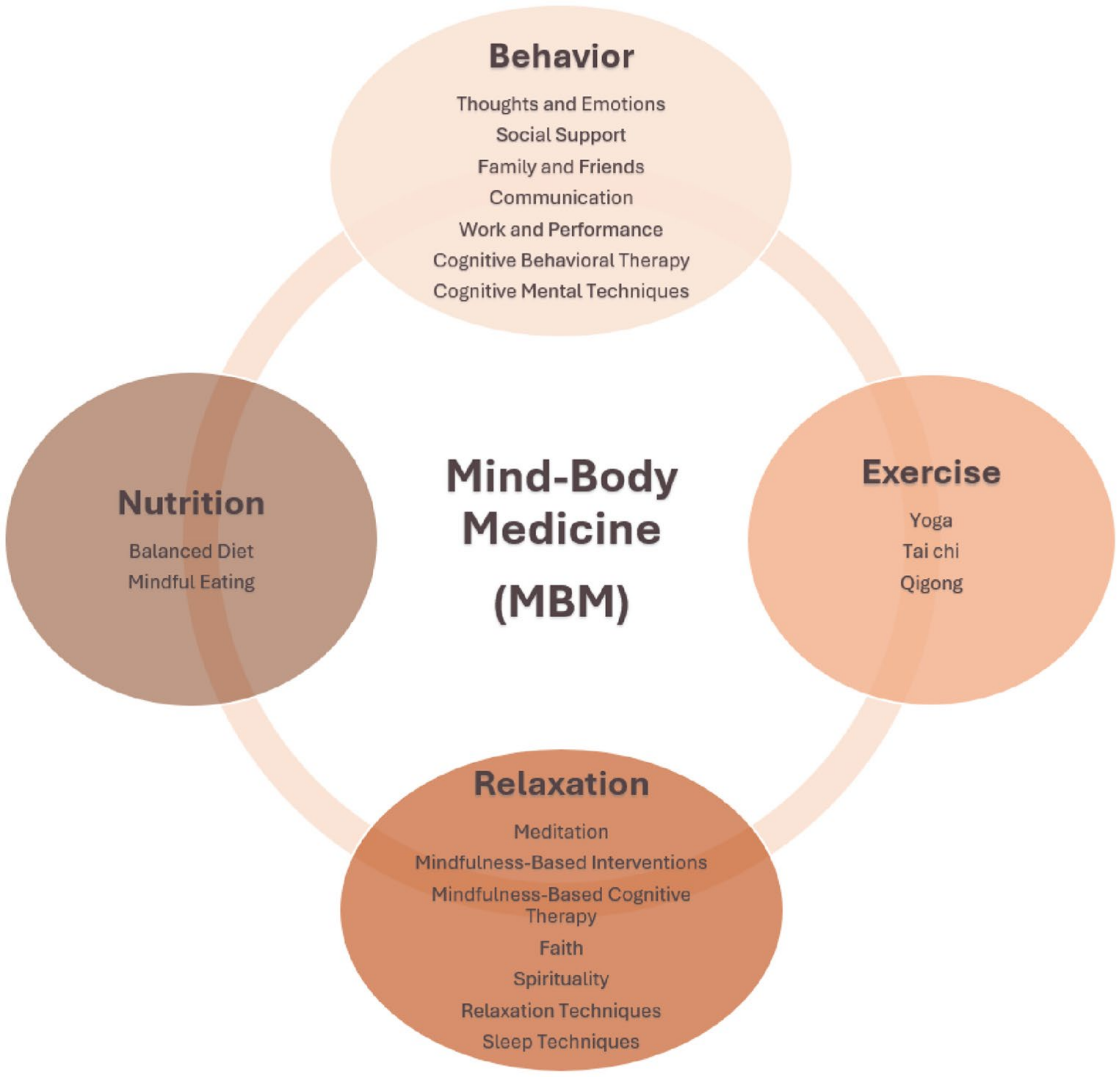
Mind-Body Medicine adapted and extended “BERN” Framework [35]

According to the National Center for Complementary and Integrative Health (NCCIH), MBM interventions are techniques that are applied or taught by a trained practitioner or teacher, aiming to facilitate the development of individual capacity for self-knowledge and self-care, to influence physical functioning by focusing on the mind, and to encourage non-judgemental acceptance of both internal (breath tracking, emotional state, etc.) and external events (smells, sounds, etc.) [40]. The “BERN” framework was developed to better understand these comprehensive multimodal therapeutic interventions and includes the following four elements: behavior (B), exercise (E), relaxation (R), and nutrition (N). The (B)ehavior pillar includes thoughts and emotions, social support, family and friends, communication, work and performance, cognitive behavioral therapy; the (E)xercise pillar includes aerobic and anaerobic exercises (yoga, tai chi, qigong etc.); the (R)elaxation pillar includes meditation, mindfulness, spirituality, faith, and sleep techniques; and the (N)utrition pillar includes a healthy balanced diet and mindful eating (see Fig. 1) [35].

Recent research has reported that the effectiveness of MBM in the treatment of psychiatric disorders, including depression, is being investigated more frequently and its use is becoming more widespread [41, 42]. It has also been reported that MBM may have less side effects than pharmacological treatment and may be cost-effective [43]. Given that depression has reached epidemic levels globally, the aim of this article is to present a review of the current evidence regarding the impact of MBM on depression. This narrative review specifically focuses on the efficacy, mechanisms, and limitations of various MBM interventions, including yoga, tai chi, qigong, mindfulness-based interventions (MBI) and nutrition. It aims to assess their potential to complement conventional treatments and their role in both symptom reduction and prevention.

## Pathophysiology of Depression

The pathophysiology of depression may depend on many different processes. Recent theories suggest that depression is associated with more complex neuromodulatory systems and neural circuits [44]. Dysregulated release of some monoamines such as serotonin, oxidative and nitrosative stress, mitochondrial dysregulation in immune cells, amygdala-frontal cortex changes, impaired opioid system and alterations of neuronal activity can be involved in the pathogenesis of depression [45]. There are also studies reporting that systemic low-grade inflammation may lead to MDD [46]. It has been reported that serum cytokine levels such as TNF-alpha, and IL-6 are increased in MDD patients, and thus the immune system is negatively affected [46]. Various studies have reported that MDD patients have increased cortisol levels with a moderate effect size, decreased hippocampus size, decreased brain-derived neurotrophic factor (BDNF) levels, and dysregulations in the HPA axis with a moderate effect size [46]. Other studies have suggested that depression is caused by disruption of homeostatic mechanisms in the brain that control synaptic plasticity, including decreased neurotrophic factors as well as reduced synaptic connections between the frontal lobe and other brain regions [47, 48].

The aetiology and pathogenesis of LLD, which is often associated with multiple comorbid physical and cognitive disorders, is complex and multifaceted [49–51]. LLD is correlated with pathophysiological mechanisms, including a decrease in brain tissue volume, alterations in vascular structure, white matter degeneration, and genetic variations [52].

The pathophysiology of multidimensional depression in the perinatal period includes biological factors such as pregnancy, which changes the hormonal balance, genetics, and growth factors [53, 54]. Increased psychosocial stress during pregnancy leads to dysregulation of the hypothalamic-pituitary-adrenal (HPA) axis and neuroendocrine hyperactivation [53]. HPA hyperactivity leads to increased cellular oxidative stress. Consequently, the immune system, neurodegeneration and neurotransmitter metabolism may be compromised, resulting in a depressed mood [18, 53].

## Potential Mechanisms and Impact of Mind-Body Medicine in Promoting Mental Health and Improving Symptoms of Depression

MBM advances mental health and well-being through the promotion of sustainable behaviours and attitudes, as opposed to the utilisation of psychological conflict elicitation techniques commonly employed in psychotherapy [35]. The salutogenic model [55] emphasises that individuals use general resilience resources to cope with stressful situations and maintain their physical and mental health through a sense of coherence [56]. This process overlaps with the approach of psychoneuroimmunology, which examines the interactions between the immune, endocrine and nervous systems. Evidence that mind-body techniques modulate immune parameters in the direction of a salutogenic profile points to both the enhancement of individuals’ ability to cope with stress and the positive impact of these skills on biological systems [57]. MBM interventions stand out as strategies to reduce the long-term negative health effects of chronic stress and are seen as specific, practical and cost-effective methods to help manage chronic stress [58].

### Yoga

Yoga is a method comprising a series of techniques, including spiritual practices, physical movements (asana), breathing exercises (pranayama), stereotyped syllables (mantras), and meditation (dhyana) [59, 60]. These techniques have their origins in Indian philosophical traditions and have been practised and developed for over 5,000 years. The objective of traditional yoga is to achieve a state of unification between the mind, body, and spirit [60]. Studies have reported that yoga provides significant benefits in terms of focusing on the present, developing self-awareness, having a positive attitude towards stress, reducing excessive increase in cortisol levels, improving self-confidence and attention [59, 61, 62].

The practice of yoga has been demonstrated to enhance an individual’s capacity to cope with stressors [63]. Practices such as pratyahara (detachment from the senses), dharana (concentration), dhyana (meditation), and samadhi (mindfulness/self-awareness) mentioned in Patanjali’s 8 steps, together with the adoption of moral principles (yama) and discipline (niyama), lead to physical and mental fitness, quality of life and improved social and environmental awareness. It can improve mood and subjective depressive symptoms and reduce the risk of depression [63, 64].

Yoga can stimulate the release of endogenous cannabinoids, endorphins, enkafalin, opiates, nitric oxide, improve endothelial function, and reduce stress hormones cortisol and catecholamines [18, 65]. In addition, yoga-based lifestyle interventions (YBI) have been reported to optimise the homeostasis of neurotransmitter substances (serotonin, dopamine, norepinephrine, acetyl-choline, GABA, glutamate etc.). These neurotransmitter substances are thought to be involved in dysregulated physiological and behavioural mechanisms in MDD [64, 66]. Optimal cellular health depends on genomic stability, oxidative stress and maintenance of telomere length. Increased oxidative stress in patients with depression may accelerate cellular ageing by impairing telomere metabolism. Previous studies show that individuals who practice yoga regularly can increase telomere length and promote cell survival by promoting BDNF and improving mitochondrial functionality [63, 64, 67]. Asanas and pranayama provide peripheral stimulation in the central nervous system (CNS), promoting BDNF levels, neuroplasticity and neurogenesis, and may produce long-term remission in MDD [64]. Yoga has been suggested to have acute and beneficial effects on heart rate variability (HRV), by having a stimulating effect on the vagal nerve, reducing activation and reactivity of the sympathoadrenal system and HPA axis. This increases parasympathetic activity and promotes relaxation and stress reduction [63, 68].

#### Yoga on Perinatal Depression

Excessive elevation of cortisol levels, which naturally increase during pregnancy, may adversely affect postnatal mental health and expose the fetus to lower birth weight and increased neurodevelopmental risks [69, 70]. Recent systematic reviews have shown that cortisol levels and depressive symptoms decreased after yoga, suggesting that yoga may regulate HPA axis activity in the short term [71, 72]. In a randomised controlled trial involving a safe yoga protocol including stretching, gentle bending, meditation, breathing exercises and relaxation techniques in the prenatal period, it was reported that pregnant women’s psychological resilience and mental well-being increased and their anxiety and depression scores were reduced [73]. Regular yoga practice in pregnant women can increase interoceptive awareness (by increasing the size of the insula) and can be empowering for stress management and overcoming fear of childbirth [74, 75].

#### Yoga on Late Life Depression

In a review of the evidence on the potential role of yoga in improving cognitive functions and mental health parameters in elderly individuals, yoga has been shown to have a positive effect on attention, executive functions and depression [76]. In five out of eight studies involving different styles of yoga, significant improvements in depressive symptoms in older adults were noted [76]. A meta-analysis on older adults with clinical depression reported that yoga and other mind-body based interventions may be more effective than aerobic and resistance exercise in improving depressive symptoms and may be associated with greater antidepressive effects [77]. This may be because MBM interventions focus on breath control, mindfulness, interoception and proprioception, which have been associated with resilience in depressive states [77].

### Mindfulness-Based Interventions

Mindfulness is the process of participating in one’s present experiences openly and without judgement [78]. It requires participants to develop a new perspective on themselves, to consciously focus on the present moment, and to approach emerging experiences without prejudice. Being constantly aware of thoughts, feelings, bodily sensations and environmental factors is the basis of mindfulness and is associated with an open, non-judgemental, accepting, curious and compassionate attitude [79]. Mindfulness enables the individual to focus on external and internal stimuli (e.g. sounds, body sensations, thoughts, emotional reactions) in their current experience. Furthermore, this state of mind involves adopting an open attitude towards one’s experiences and encompasses both a tendency for people to increase their level of mindfulness in everyday life and practices that promote mindfulness [80]. While mindfulness has its roots in Buddhist culture, mindfulness-based interventions (MBI) are an umbrella term for a range of mindfulness-centred, non-religious psychological interventions such as mindfulness-based stress reduction therapy (MBSR) or mindfulness-based cognitive therapy (MBCT) [81, 82].

Some systematic reviews examining the efficacy of MBI for depression and anxiety have reported that mediators such as decentering, acceptance, and mindful attention may help alleviate stress mechanisms [83]. In particular, it has been shown that patients with depressive moods experience less rumination and move away from negative thought patterns as their decentering skills improve [68]. MBI can facilitate coping with stressors associated with depression, help them better recognize and distance themselves from maladaptive thought patterns, thus reducing subjective symptoms [68].

Several brain areas affected by depression, such as the prefrontal cortex, anterior and posterior cingulate cortices, parietal cortex, basal ganglia, can be modulated by mindfulness practices (breath field, body scan, voice scan, etc.) [78]. It can therefore be concluded that it has a direct positive effect on the brain. In addition, many stress markers associated with depression such as C-reactive protein, cortisol, TNF-alpha, heart rate and systolic blood pressure can be regulated by mindfulness techniques, thus reducing negative emotional responses and increasing subjective well-being [78]. The increase in antibody concentration, a marker of immune recovery, may be induced by increased activity in the left prefrontal lobe in individuals practising mindfulness meditation [54]. MBCT, which combines mindfulness and cognitive practices, is particularly common among individuals with major depression and may reduce the recurrence of depression [84]. MBCT, which aims to control negative thought patterns that lead to exacerbation of symptoms in individuals with major depression, has been shown to be effective in reducing relapse rates [85]. It has also been reported to improve symptoms of acute depression in individuals with MDD [86]. The results of the meta-analysis indicated that MBI had a moderate positive effect on self-reported awareness [85]. Therefore it can facilitate a more objective perspective on the events experienced, as well as improved emotional regulation and reactions [85, 87]. The combination of behavioural activation with mindfulness has been demonstrated to elicit both short- and long-term reductions in depressive symptoms among patients with subthreshold depression [88].

In a recent meta-analysis, it was reported that, overall mindfulness interventions compared to active and passive control groups are moderately effective in reducing depressive symptoms in the elderly, although there are very few studies examining the effect of MBI in older adults [89]. In a meta-analysis investigating the effect of MBI on menopausal women, it was shown that MBI significantly reduced stress scores, promoted coping with stress, and reduced repetitive negative thoughts. However, it showed that mindfulness interventions had no significant effect on depression, anxiety or mindfulness scores in menopausal women compared to passive and active control groups [81].

Mindfulness is included in the concept of meditation and there are many different meditation techniques. However, different forms of meditation and their effects on depression have not been examined extensively in the literature. In a meta-analysis examining the effects of mantra-based meditation on mental health, small but significant improvements in depression and mental health-related quality of life were reported. There was also no significant difference between the effectiveness of mantra-based meditation and psychotherapy in promoting potential mental well-being [74].

#### Mindfulness on Perinatal Depression

MBI were shown to be effective in reducing perinatal depressive symptoms, however the role of symptom intensity is still unclear. In one meta-analysis, MBI were more effective in perinatal depressed women with moderate and severe symptoms than in those with minimal and mild symptoms [90]. However, in another systematic review, MBI were shown to improve postnatal depression better in pregnant women with subthreshold depression than in pregnant women with severe depressive symptoms [91]. Although there are other studies showing that mindfulness meditation can effectively alleviate and control negative emotions during pregnancy and reduce the incidence of depression [54, 91, 92], there are also meta-analyses reporting inconsistent results in reducing perinatal depression. It is possible that non-compliance with the intervention and high dropout rates may explain these inconsistent findings [78].

#### Mindfulness in Adolescent and Young Adult Depression

While it may take some time for depressed adolescents to develop socio-emotional skills, get along well with peers and teachers, and take responsibility, they can adapt easily and depressive symptoms may improve, especially during middle adolescence due to increased brain plasticity [93, 94]. Mindfulness interventions are effective in reducing mild to moderate depressive symptoms in adolescents aged 10–19 years, however the type of mindfulness intervention, individual counselling and length of follow-up were shown to moderate the effect size [93, 95]. Previous studies investigating whether MBI have a significant effect on depression, stress, sleep quality, anxiety, mindfulness scores, and academic achievement have reported positive benefits of MBI on depression symptoms of young adults [95–98].

### Tai-Chi and Qigong

Tai chi, one of the traditional Chinese exercises, was originally developed as a martial art [99]. Over time, however, it has become a popular approach to exercise, utilising conscious movement, deep rhythmic breathing techniques and meditation techniques to achieve mental and physical harmony [100].

Qigong is a set of practices dating back more than two thousand years ago, using the therapeutic effects of components such as concentration, relaxation, proprioceptive and interoceptive awareness, meditation, breath regulation and gentle movements [101, 102].

Current evidence shows that tai chi and qigong practices may have positive effects on reducing subthreshold and major depression levels and lifelong depression [103, 104]. Tai chi and qigong practices can improve psychological well-being, self-esteem, self-awareness, concentration, reduce negative reactions and improve symptoms of depression [100, 105]. In terms of psychological mechanisms, tai chi and qigong can improve mental health by improving self-efficacy. It provides social support by being experienced with the community and instructs the individual to calm the mind and let go of negative emotions through mindfulness and imagery practices [106, 107]. As an example of imagery in tai chi, techniques such as “cloud hands”, “white crane spreading its wings”, and “separating the mane of a wild horse” can reduce depression by increasing positive emotions and diverting attention [106]. In a recent study, it was pointed out that some exercise approaches, including tai chi, may be effective in reducing and preventing depression symptoms of college students, increasing students’ concentration, self-awareness, self-efficacy and consequently alleviating depression [108]. According to possible physiological mechanisms, the practice of tai chi and qigong may be associated with lower levels of IL-6, CRP and salivary cortisol [103, 106]. In addition, it may show anti-depressant effects by increasing the levels of anti-inflammatory factors such as IL-10 [109]. It may also show improvement in parameters that are markers of parasympathetic and sympathetic nervous system balance, such as increased HRV [103, 106]. By relaxing the body and mind, parasympathetic nerves are stimulated, heart rate slows, blood pressure decreases, peripheral sensory functions are stimulated and the endocrine system is regulated. Vagal nerve function is increased and negative psychological symptoms are alleviated [103, 107, 109]. Furthermore, tai chi and qigong can promote the release of neurotransmitters such as dopamine, serotonin and adrenaline in the brain; positive mood can promote the release of endogenous opioids such as beta-endorphins [103, 104, 110]. They may promote neuroplasticity by improving BDNF levels while enhancing the concerted function of the limbic system and prefrontal cortex associated with emotional regions in the brain. Given the function of the prefrontal cortex in mediating emotions, increasing the thickness of grey matter in the prefrontal cortex may elicit promising responses in coping with psychological disorders such as depression [100, 105, 106].

In a meta-analysis investigating the effects of various traditional chinese exercises (tai chi, baduanjin, wuqinxi, yijinjing and liuzijue) on depression, tai chi was reported to be an appropriate intervention for the treatment of depression [111]. In a study investigating the effects of exercises such as yoga, tai chi, dance, running, running, basketball, badminton, and volleyball on depressed college students, it was reported that the most effective intervention in reducing depression was tai chi, followed by yoga. It was concluded that if these practices include not only exercise but also meditative techniques, they may encourage a greater reduction in depressive symptoms [112]. Tai chi and qigong practices showed moderate to high efficacy in alleviating depression symptoms in elderly people without mental disorders [113]. Another meta-analysis shows that they are effective in improving adolescents’ mental health and reducing anxiety and depression, but qigong improves depressive symptoms more than tai chi. It is thought that this may be because qigong focuses on internal energy flow, the mind, whereas tai chi focuses on environmental stimuli, defence and offensive intentions [114]. Tai chi and qigong may be more effective than yoga in alleviating depression and improving psychological quality of life in older adults (as the exercises in yoga are more physically demanding) [113]. However, qigong in particular may offer psychological benefits beyond biological mechanisms and may be recommended as a safe, easy-to-learn, clinically effective rehabilitation method [107].

## Nutrition and Mindful Eating for Depression

As mentioned above, MBM-based interventions follow multimodal strategies that have an impact on behavior, exercise, relaxation, and nutrition. So, a well-balanced diet is one of the main principles in MBM [35]. A growing body of research investigated the positive effect of a healthy Mediterranean diet on depression [115–117]. Mediterranean diet is characterized by a high intake of fruits, vegetables, legumes, grains, nuts, fish, and olive oil and low intake of meat and dairy products [118, 119]. The effects of the Mediterranean diet are reflected in a current meta-analysis displaying a significant moderate effect on depression severity [119]. These potential benefits can be attributed to anti-inflammatory [120] and anti-oxidative properties [121], providing enough mood-regulating B vitamins which are crucial to neurotransmitter production, as well as vitamin D and omega-3 fatty acids which protect against pro-inflammatory cytokines [122]. A recent systematic review found a negative association between polyphenol consumption in Mediterranean diet and depression risk, and also evidence suggesting polyphenols can effectively alleviate depressive symptoms [123]. BDNF concentrations could also be influenced by Mediterranean diet interventions. In a clinical trial, patients which followed a Mediterranean diet pattern were less likely to have lower BDNF levels, and even after 3 years of intervention, a statistically significant increase in BDNF was observed in comparison to the control group [124]. Another pathway to influencing mental health through nutrition relates to the way food is consumed, such as mindful eating. This focuses on sensory elements of eating and a non-judgmental awareness of thoughts and feelings while eating, awareness of hunger and satiety, finding a quiet place and avoiding distractions [125, 126]. A direct positive effect of mindful eating on depression is shown in a mediator analysis [127], and a meta-analysis shows a positive effect of mindfulness interventions on depression in overweight people [128]. Further, potential benefits of mindful eating on eating behaviors such as binge eating, emotional eating and eating in response to external stimuli are described [128, 129]. Mindful eating also plays an important role in weight loss, especially in maintaining weight loss [128], which is interesting considering the strong correlation and dose-response relationship of obesity and depression [130, 131].

## Safety Considerations and Adverse Events

Studies show that the side effects of MBM interventions in patients with depression are mild and generally limited to musculoskeletal pain, while serious adverse events are rare [132, 133]. MBM interventions have a high safety profile for patients with depression and carry a comparable risk of adverse events to other physical activities [77, 133]. In a systematic review examining the adverse effects of meditation-based practices, the prevalence of adverse events associated with meditation practices was 8.3%, which is similar to that of other psychotherapy practices [132]. Adverse events have been reported to occur mostly during or immediately after meditation practice or intervention and include depression, anxiety, psychotic or delusional symptoms, fear, suicidal ideation and suicidality [132, 134]. However, it has been observed that such events are under-reported in the literature, especially in randomised controlled trials [135]. Taking into account that all MBM practices are meditation-based, any adverse events that may occur in individuals with depression should be carefully evaluated and thoughtful consideration is required when working with clinical samples. It is important that MBM is not applied as an alternative treatment, but within an integrated care model to prevent worsening of symptoms and suicide risk [136]. It is also important to consider whether referral to MBM therapies, particularly in cases of severe depression, may require a referral from mental health providers. Open and ongoing communication between MBM providers and mental health professionals, follow-up of patients, and multi-disciplinary collaboration can play a critical role in ensuring patient safety and minimising risks. The participation of MBM providers in adequate training, licensing, mentoring and sustained support programmes is critical for therapy efficacy and patient safety. Furthermore, inadequate reporting of adverse events in previous randomised controlled trials may limit the translation of these risks into clinical practice. Therefore, more studies are needed to clarify adverse events.

## Conclusion and Future Directions

According to this narrative review, mind-body medicine appears to be a potentially effective and beneficial intervention in reducing depression symptoms. MBM interventions are accesible, easy to implement, cost-effective and can be integrated with primary treatments. However, due to high heterogeneity and some methodological concerns, caution is required when interpreting and generalising findings regarding the efficacy of MBM on depression. There is no concluding evidence regarding the optimal frequency and duration of MBM interventions required to improve symptoms of depression.

Although there is evidence that yoga improves mental and physical well-being and has antidepressant effects, more studies investigating its long-term effects in the prevention and treatment of depression are needed [104, 133]. Recent meta-analyses have reported inconsistent findings regarding the effects of yoga interventions on depression; these inconsistencies may be due to methodological limitations in study design, data reporting, and intervention definitions [62, 133]. Similarly, Tai Chi shows a medium-high effect size in reducing stress, but there is insufficient evidence to conclude on its effectiveness on depression [137]. Moreover, qigong can be promoted as a preventive, complementary intervention for depression, but more well-designed RCTs are needed to draw firm conclusions and validate the evidence for its effect on depression [138].

MBM can be used in combination with conventional therapies to help manage many psychological disorders and improve overall well-being, in both inpatient and outpatient psychiatric clinics with a multidisciplinary approach to improve person-centred mental health services [139]. Table 1 outlines some practical considerations for an integrated MBM approach. MBM therapies can be recommended and followed by different health professionals such as physicians, psychologists, physiotherapists, dietitians and MBM providers complementary to psychiatric treatment. This integrated model may reduce the stigma attached to mental health illnesses, which in turn may lead to greater use of mental health services by both clients and caregivers [139]. Referral processes should be based on the severity of depression as defined by health professionals, the patient’s response to treatment and possible risk factors. For instance, Tai chi and qigong may be appropriate as complementary treatment for patients receiving pharmacotherapy who are willing to undergo behavioural intervention, but these practices should not replace conventional medical treatment when the assessment of a qualified mental health professional is required [140]. People with severe impairments in cognitive functioning (e.g. psychosis) may not be suitable candidates for mindfulness training, and patients experiencing dissociation may be at risk of having their symptoms increased during mindfulness practice [140].

**Table 1:** Practical considerations for an Integrated MBM Model

Referral	Selection of appropriate MBM treatment according to the severity of depression by multidisciplinary health professionals and MBM providers.
Indication	Psychological disorders such as mild to moderate depression, stress management, anxiety disorders, sleep problems. Gradual or controlled counselling in cases of severe depression.
Contraindication	In severe mental conditions such as active psychotic symptoms, suicide risk, dissociative disorders, MBM should be applied with caution and require close follow-up.
Follow-Up	Recording the duration and frequency of MBM and side effects and following up the treatment plan with electronic medical records.
Clinical Assessment Tools	Use a variety of assessment tools to establish a common language between health professionals, both among themselves and with MBM providers. Beck Depression Inventory (BDI), Hamilton Depression Rating Scale (HDRS or HAM-D), Patient Health Questionnaire-9 (PHQ-9), Geriatric Depression Scale (GDS), Edinburgh Postnatal Depression Scale (EPDS), Depression Anxiety Stress Scales (DASS), etc.
MBM and Standard Treatment Sustainability	Supporting individual care with digital platforms, mobile applications and online monitoring tools to ensure sustainability of MBM and standard treatment.

MBM = Mind-Body Medicine

In the future, the integration of these therapies is expected to expand, particularly as evidence continues to grow regarding their effectiveness in enhancing clinician-assessed and self-reported depressive symptoms [141]. There is also an increasing focus on personalised approaches that combine MBM in digital environments with technological innovations such as mobile apps, online courses and workshops, web-based tools and platforms to support long-term mental health management [142–144]. In the future, if these technological applications prove to be easily accessible, scalable and cost-effective, their use to improve mental health may become even more popular and promote well-being among the general population.

## Electronic Supplementary Material

Below is the link to the electronic supplementary material.

Supplementary Material 1

Supplementary Material 2

Supplementary Material 3

Supplementary Material 4

Supplementary Material 5

## Data Availability

No datasets were generated or analysed during the current study.
